# Analysis of LCT-13910 genotypes and bone mineral density in ancient skeletal materials

**DOI:** 10.1371/journal.pone.0194966

**Published:** 2018-04-30

**Authors:** Barbara Mnich, Anna Elżbieta Spinek, Maciej Chyleński, Aleksandra Sommerfeld, Miroslawa Dabert, Anna Juras, Krzysztof Szostek

**Affiliations:** 1 Department of Anthropology, Jagiellonian University, Cracow, Poland; 2 Department of Anthropology, Polish Academy of Sciences, Wroclaw, Poland; 3 Institute of Archaeology, Adam Mickiewicz University in Poznan, Poznan, Poland; 4 Department of Agriculture and Bioengineering, University of Life Sciences, Poznan, Poland; 5 Molecular Biology Techniques Laboratory, Adam Mickiewicz University in Poznan, Poznan, Poland; 6 Department of Human Evolutionary Biology, Adam Mickiewicz University in Poznan, Poznan, Poland; Garvan Institute of Medical Research, AUSTRALIA

## Abstract

The relation of LCT-13910 genotypes and bone mineral density (BMD) has been the subject of modern-day human population studies, giving inconsistent results. In the present study we analyze for the first time a relation of LCT-13910 genotypes and BMD in historical skeletal individuals. Ancient population might be a model for testing this association due to elimination of non-natural factors affecting bone density. Among 22 medieval individuals from Sanok churchyard (South-Eastern Poland; dated from XIV to XVII c. AD) we identified 4 individuals with osteoporosis (mean BMD = 0.468 g/cm^2^, SD = 0.090), 10 individuals with osteopenia (mean BMD = 0.531 g/cm^2^, SD = 0.066) and 8 individuals with normal BMD values (mean BMD = 0,642 g/cm^2^, SD = 0.060). Analyses of BMD and LCT-13910 genotypes revealed that mean BMD was the highest (0.583 g/cm^2^, SD = 0.065) in the individuals with lactose tolerance genotypes (TT and CT). We also found possible association of lower BMD at the radius and CC genotypes due to higher but not statistically significant frequency of osteoporosis in the lactose intolerant group (*p* = 0.60). Statistically significant correlation was found between BMD and females aged 20–35 years, with tendency to reduce BMD with age (*p* = 0.02).

## Introduction

Lactose intolerance (LI) is inherited as an autosomal recessive trait which leads to the down-regulation of lactose-phlorizin hydrolase (LPH) enzyme activity, in the intestinal cells, what decreases the ability to convert lactose into absorbable sugars of glucose and galactose after weaning [1]. The decline of LPH enzyme activity is supposed to occur by the age of 12 years, however a part of people still retain their neonatal LPH activity displaying lactose tolerance (LT) [2]. It was shown that differences in LPH expression in adults correlate with LCT-13910 C/T (rs4988235) polymorphism located in the intron 13 of the *MCM6* gene [3].

One of the major debated issues connected with lactose is the relation of LI with bone density and the risk for fractures and osteoporosis. Although the influence of LI on bone mineral density (BMD) and calcium intake were discussed in recent decades, this issue still remains unclear. Most of clinical studies focused on postmenopausal women and elderly men. In result, several authors reported significant association of LCT genotypes and BMD [4–6], while others have failed to confirm this relation [7, 8]. It was also observed that LI/LT influenced reaching the peak of bone mass (PBM) in young adults [4].

It was reported that LCT-13910 CT and TT genotypes associated with LT were subjected in Europe to a strong positive selection for over 3000 years. The TT and CT genotypes were absent in early Neolithic Farmer populations and present in only 10% of Bronze Age European people [9, 10, 11]. In Europe the LT has increased from Iron Age through Medieval Period till present day times [12]. Nowadays the frequency of LI in Europe, represented by LCT-13910 CC genotypes, varies from 2% in Scandinavia to 20–40% in Central Europe [5] and to 60–70% in Italy and Turkey [13].

We assume that ancient population might be a good model for testing association of BMD and LI, due to elimination other than natural and physiological factors which may influence bone density. Milk was one of the main sources of calcium, at the time, what could make the possible relation between LCT-13910 genotypes and BMD more prominent. Here we present for the first time the relation of LCT-13910 genotypes and BMD in ancient human population and the possible association of BMD and age of women in reproductive period.

## Materials and methods

### Materials

**Fig 1:**
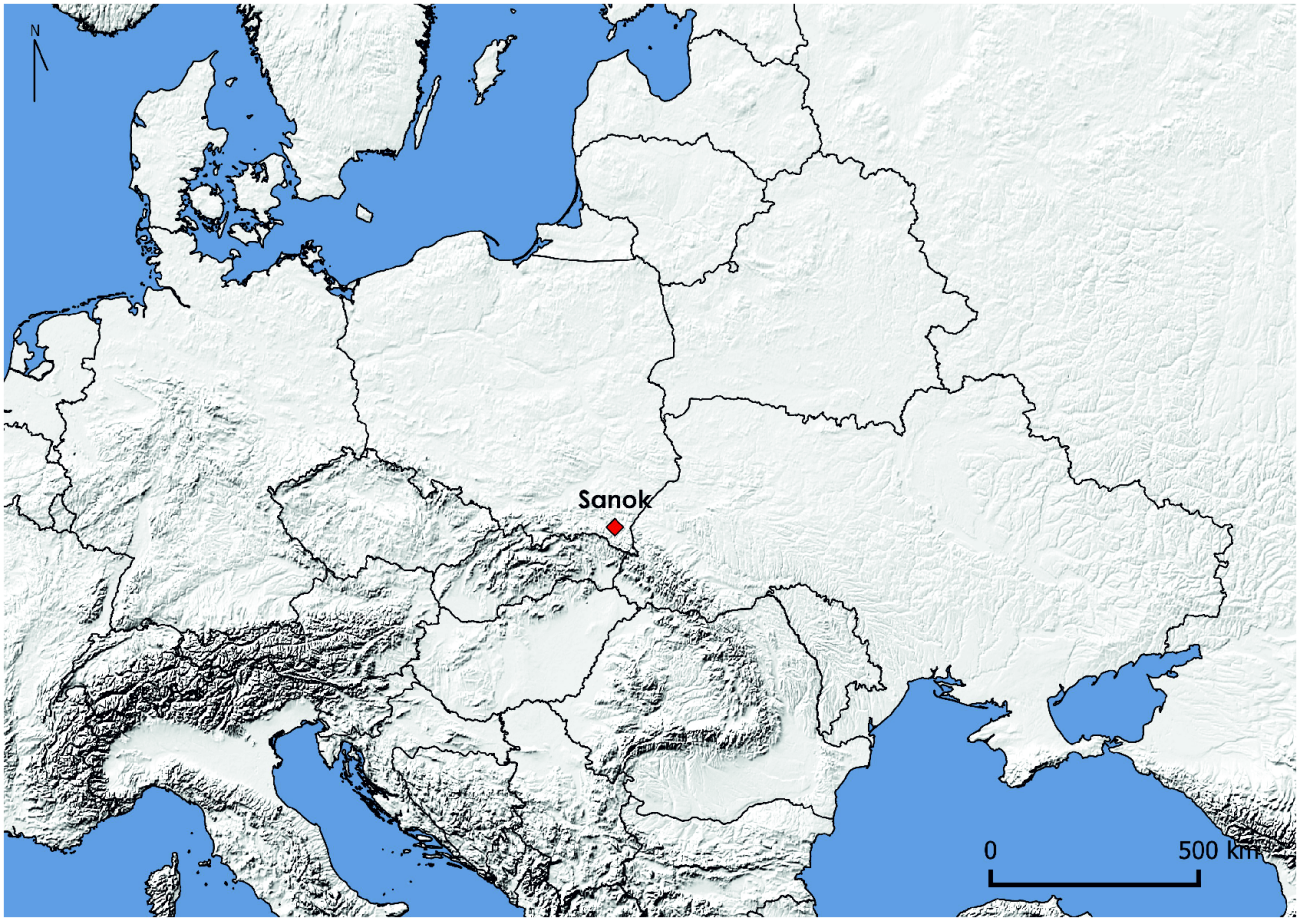
Localisation of Sanok burial site.

Analyses were conducted on 22 medieval individuals (10 females, 12 males) from Sanok churchyard (South-Eastern Poland, Podkarpackie region) (Fig 1), dated from XIV to XVII c. AD, in age at death between 24 and 60 years. The sampling of bone materials from the Anthropological Collection at the Jagiellonian University in Cracow was permitted by Professor Krzysztof Szostek (permission number 003/2017). The morphological description of each individual is presented in S1 Text. BMD was measured in all individuals while only 17 were available for genetic analyses. DNA was extracted from teeth or petrous bones. The morphological sex and age-at-death of the individuals were assessed through standard methods used in physical anthropology [14].

### Bone mass density determination

To determine BMD, we used fully preserved right or left radius with fused epiphyses and with no signs of soil erosion. The bones were measured by Dual X-Ray Absorptiometry (DXA) using Hologic Discovery W densitometer (Hologic Inc., Bedford, MA) at the Medical University Hospital in Wroclaw, Poland. DXA allowed to measure bone mineral content (BMC) using absorption data of two different length X-ray beams what led to determine bone mineral density (content of hydroxyapatite in bone) in g/cm^2^ according to [15]. This technique is commonly used for estimating of BMD of skeletal remains [16]. To compensate for the lack of soft tissues, the bones are placed in, for instance, dry rice [17, 18]. In this study, radial bones were put in a container filled with rice at a depth of 3–4 cm. BMD was measured in the distal radius region (on 1/3 distal part of radius bone). Due to the lack of ancient data, we used the reference modern data provided by the manufacturer of Hologic Discovery W densitometer, as a part of the software. Our BMD results were showed as T-score values which are a deviation from the normal mean value established for healthy young Caucasian adults in SD unit [4]. Norm (N) was defined as a T-score higher than -1 SD, osteopenia (OPN) as a T-score between -2.5 and -1 SD, and osteoporosis (OP) as a T-score less than -2.5 SD [19, 20]. All criteria used for the diagnosis of osteoporosis were defined by the World Health Organization (WHO), the National Institute of Health (NIH) and International Society for Clinical Densitometry (ISCD) and have been used to diagnose osteopenia and osteoporosis in other skeletal remains [18, 21, 22].

### Ancient DNA extraction and next generation sequencing

Teeth or petrous bones were collected using disposable gloves and mask in order to minimize the risk of modern DNA contamination. DNA extractions were conducted in the sterile laboratory dedicated to ancient DNA analyses, at the Faculty of Biology, Adam Mickiewicz University in Poznan (AMU), Poland. Samples were cleaned with ~ 5% NaOCl, rinsed with sterile water and UV irradiated as described before [23]. Roots of the teeth and the inner ear part of petrous bones were drilled with the use of Dremel^®^ drill bits. ~200 mg of bone powder was collected to the sterile tubes and DNA was extracted as described previously [23]. A 52 bp fragment comprising LCT-13910 was amplified using LCTF (GAGAGTTCCTTTGAGGCCAG) and LCTR (GCGCTGGCAATACAGATAAG) primers fused with barcodes and Ion Torrent adapters. For each DNA extract, 2–3 separate PCR reactions were set up using AmpliTaq Gold 360 Master Mix (Life Technologies), 0.2 μM each primer, and 3 μl of DNA template. PCR conditions were as follows: 2 min at 94°C, 40 cycles of 1 min at 94°C, 30s at 60°C, 1 min at 72°C and final extension at 72°C for 5 min. Negative controls were set up during extractions (one control for every eight extractions) and amplifications (one negative control for every eight PCR reactions). Pooled PCR products were separated by 2% agarose gel electrophoresis and purified with QIAquick^®^ Gel Extraction Kit (Qiagen). Fragment length distribution and concentration of libraries were measured with the use of 2200 TapeStation system (Agilent). Sequencing was conducted on high-throughput PGM Ion Torrent system using Ion 314^™^ Chip Kit v2 and the Ion PGM Hi-Q sequencing kit v2 following the manufacturer’s instructions.

### Determination of LCT-13910 genotypes

Sequencing data were processed using a custom analytical pipeline. The fastx_barcode_splitter.pl script (http://hannonlab.cshl.edu/fastx_toolkit/) was used to separate reads by barcodes using a one mismatch threshold. Cutadapt v1.8.1 software was applied to trim the adapters [24]. BWA aln version 0.7.8 [25] with the non-default parameters -l 16500 -n 0.01 -o 2 -t 2 was used to map the reads against the reference human genome (built 19). Genotyping results and coverage at LCT-13910 locus were visualized with Biomatters IGV software v2.3.66 [26]. The proportion of the non-reference allele between 20% and 80% was identified as heterozygous genotype; otherwise, homozygous genotype was called as described by [27].

### Statistical analyses

Linear regression was used to determine correlation between BMD and age of females (aged 20–35). The differences between LI and LT groups in OP classification were tested by Fisher’s exact test. Non-parametric version of ANOVA (Kruskal-Wallis H test) was used to test the heterogeneity in BMD between different genotypes (TT, CT, CC). The statistical analyses were performed in Python (3.5.0) with the help of SciPy library (0.18.1) [28].

## Results

**Fig 2:**
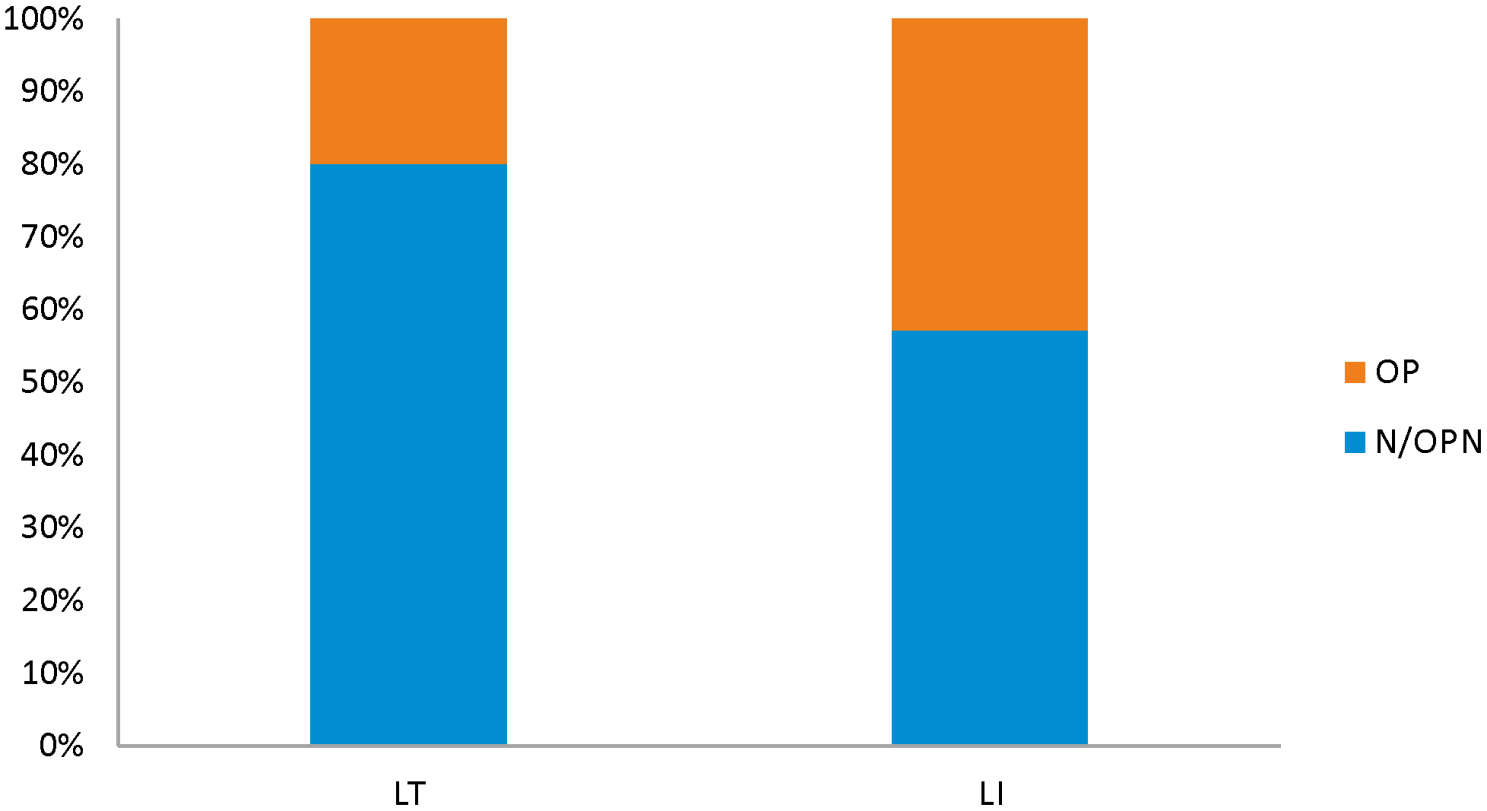
Correlation between BMD and age among medieval females (*p* = 0.02).

Analyzed BMD values determined for each out of 22 ancient individuals varied from 0.338 g/cm^2^ to 0.683 g/cm^2^, with mean value of 0.560 g/cm^2^ (SD = 0.094) at the radius bone (Table 1). Mean value of BMD was 0.603 g/cm2 (SD = 0.075) and 0,508 g/cm^2^ (SD = 0.089) for males and females, respectively. Among analyzed individuals, all BMD classes (N, OPN and OP) have been assigned based on T-score values. We identified 4 individuals with OP (mean BMD = 0.468 g/cm^2^, SD = 0.090), 10 individuals with OPN (mean BMD = 0.531 g/cm^2^, SD = 0.066) and 8 individuals with N (mean BMD = 0,642 g/cm^2^, SD = 0.060). Using linear regression, statistically significant correlation was found between BMD and age of females (aged 20–35 years) with tendency to reduce BMD with age (r = -0,75, r^2^ = 57%, *p* = 0.02) (Fig 2).

**Table 1:** Characteristics of medieval individuals according to their age at death, sex, BMD values, T-score values, LCT -13910 genotypes and number of sequence reads for C and T alleles.

ID	Age at death	Sex^a^	R/L^e^ radius	BMD Total (g/cm^2^)	T-score values	BMD class	LCT-13910 genotype	No. of sequence reads for allel C	No. of sequence reads for allel T	LT/ LI^d^
**25**	45–49	M	L	0.565	-2.3	OPN	CT	22139	24116	LT
**34**	30–35	F	R	0.460	-2.2	OPN	CC	6280	15	LI
**41**	50–55	M	R	0.517	-3.3	OP	CT	1088	891	LT
**47**	25–29	F	R	0.488	-1.7	OPN	n.a.	n.a.	n.a.	n.a.^b^
**63**	25–28	M	R	0.683	-0.1	N	n.r.	n.r.	n.r.	n.r.^c^
**64**	43–47	M	R	0.536	-2.9	OP	CC	24551	17	LI
**95**	23–26	F	R	0.629	0.9	N	n.a	n.a.	n.a.	n.a.
**96**	40–50	M	R	0.482	-4	OP	CC	820	0	LI
**99**	40–45	M	R	0.594	-1.8	OPN	CC	8290	48	LI
**103**	40–47	M	R	0.676	-0.2	N	n.r	n.r.	n.r.	n.r.
**124**	30–35	F	R	0.491	-1.6	OPN	n.a.	n.a.	n.a.	n.a.
**125**	24–26	F	R	0.600	0.4	N	CC	12675	281	LI
**130**	28–30	F	R	0.607	0.5	N	CT	7310	6113	LT
**146**	35–45	M	L	0.602	-1.6	OPN	TT	687	22724	LT
**161**	35–40	M	R	0.564	-2.4	OPN	TT	112	7857	LT
**168**	35–40	M	R	0.730	0.8	N	n.r	n.r.	n.r.	n.r.
**198**	25–30	F	R	0.539	-0.7	N	n.a.	n.a.	n.a.	n.a.
**208**	55–60	F	R	0.432	-1.7	OPN	CT	15032	17007	LT
**220**	30–35	M	L	0.623	-1.1	OPN	CC	12659	13	LI
**222**	35–40	M	L	0.668	-0.4	N	n.r.	n.r.	n.r.	n.r.
**243**	30–40	F	L	0.338	-4.5	OP	CC	10921	298	LI
**256**	35–44	F	R	0.492	-1.6	OPN	n.a.	n.a.	n.a.	n.a.

^a^M—male; F—female

^b^n.a.—not analyzed

^c^n.r.—no result (unsuccessful amplification of LCT-13910 locus)

^d^LT—lactose tolerant; LI—lactose intolerant

^e^R—right; L—left

**Fig 3:**
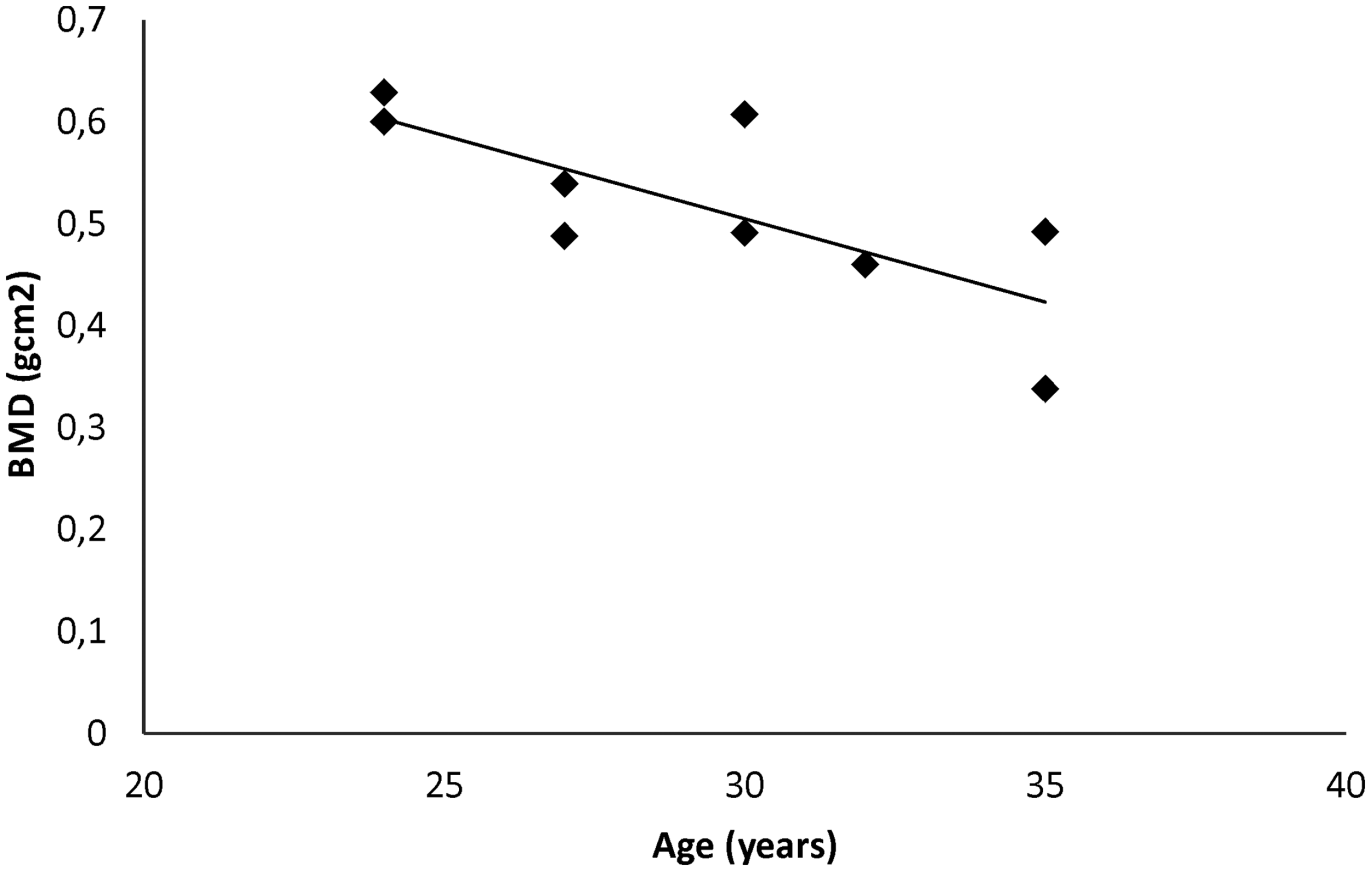
The percentage of individuals from particular BMD class (OP and N/OPN) among LI and LT group.

LCT-13910 genotypes were successfully determined in 13 individuals (Table 1). The distribution of retrieved genotypes was as follows: 2 individuals with LCT-13910 TT, 4 individuals with LCT-13910 CT and 7 individuals with LCT-13910 CC (Table 1, Fig 3). For 4 individuals (sample nos. 63, 103, 168 and 222) we were not able to establish LCT genotypes due to unsuccessful PCR amplification. DNA sequence data were deposited in NCBI Sequence Read Archive (SRA) under accession number SRP130254.

Comparisons of BMD and LCT revealed that mean BMD was the highest in LT individuals with LCT-13910 TT genotypes (mean BMD 0.583 g/cm^2^, SD = 0.027) and LCT-13910 CT (mean BMD 0.530 g/cm^2^, SD = 0.075). The lowest values were observed in LCT-13910 CC genotypes (mean BMD 0.519 g/cm^2^, SD = 0.101). Female with the lowest BMD (sample no. 243) was found in LI group. Among LT group only one individual with OP was identified (sample no. 41), while three individuals with OP were found (samples nos. 243, 64 and 96) among LI. According to Fisher’s exact test the distribution of OP in LI and LT was not statistically significant (*p* = 0.60). The heterogeneity in BMD between different genotypes (TT, CT, CC), analyzed with the use of Kruskal-Wallis H test, was also not statistically significant (*p* = 0.73).

## Discussion

This is the first study of BMD and LCT-13910 genotype relations that was performed on an ancient population. Although the number of analyzed individuals is low when compared to modern population studies, we found possible association of lower BMD and LI, at the radius. We noted higher, but not statistically significant frequency of OP in LI group (*p* = 0.60). Although there are no ancient comparative data on BMD and LCT-13910 genotypes, our data are consistent with previously reported results acquired for radius, total hip and the Ward’s triangle BMD in a present-day postmenopausal Caucasian women [6] or for lumbar and femoral neck BMD in a postmenopausal women from Austria [5]. The association of lower BMD and LCT-13910 CC genotype has been also indicated in the studies of elderly people [29] and recently in the modern children from Malay in Malesia [30].

Among LT individuals we found one adult man with OP, aged 50–55 years (sample no. 41). The appearance of OP in this individual could be the effect of an age related bone loss or the idiopathic osteoporosis connected with some secondary cause, which is also observed in present-day patients [20]. Notably, in LI group the age of individuals with OP was quite low and established as 35–45 years (samples nos. 243, 64 and 96). Early pathological bone loss might be then explained by reaching a lower peak bone mass (PBM) in adolescence due to a lower calcium intake caused by LI. Interestingly, 35 year old female with OP (sample no. 243) might be assumed to be during or just after her reproductive period [31]. Because PBM is usually reached approximately at the age of 30 [15], BMD should be then the highest at her age. However, it was shown that long lasting breastfeeding causes increased calcium absorption from bones and leads to periodic BMD decrease [31]. Thus we might suppose that both, LI and breastfeeding could lead to osteoporotic bone mineral density values which occurred in the analyzed female. Moreover, Turner-Walker and Mays [31] claimed that in medieval females, in age between 22 and 35 years, BMD decreased. Although the strength of this tendency might vary between populations due different pregnancy patterns, we confirmed this correlation in our medieval females, since significant negative correlation between age and BMD was identified (in age group between 20 and 35 years).

In conclusion, our data from historical medieval individuals suggest possible association of LCT-13910 CC and lower BMD; however, we cannot exclude samples bias due to small population size. Past population could be treated as a model for verification of LCT-BMD association, although to get a better understanding of this relation, future studies should include more ancient individuals from different periods of time.

## Supporting information

S1 Text Preservation of human skeletons used in the study. (DOCX)
