# Correction:Analysis of LCT-13910 genotypes and bone mineral density in ancient skeletal materials

**DOI:** 10.1371/journal.pone.0236908

**Published:** 2020-07-23

**Authors:** Barbara Mnich, Anna Elżbieta Spinek, Maciej Chyleński, Aleksandra Sommerfeld, Miroslawa Dabert, Anna Juras, Krzysztof Szostek

There is an error in affiliation 2 for author Anna Elżbieta Spinek. The correct affiliation 2 is: Department of Anthropology, Ludwik Hirszfeld Institute of Immunology and Experimental Therapy, Polish Academy of Sciences, Wroclaw, Poland.
