# Supplementary material for: Analysis of LCT-13910 genotypes and bone mineral density in ancient skeletal materials
Source: PLoS One. 2018 Apr 30;13(4):e0194966. doi: 10.1371/journal.pone.0194966 (PMC5927400; doi:10.1371/journal.pone.0194966)
Supplement: S1 Text — (DOCX) [file pone.0194966.s001.docx]

**Supplementary Text**

**Preservation of human skeletons used in the study**

**Individual no. 25 (male, 45-49 years)**


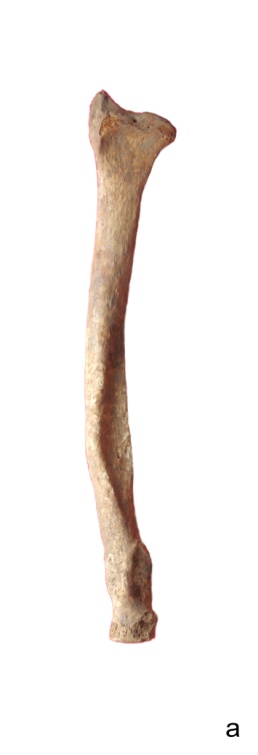
A medium-preserved skeleton. Skull bones were present, except maxilla. Mandible with few teeth was present. The postcranial skeleton was represented by the following bones or their fragments: clavicles, scapulas, sternum, ribs, humeri, ulnae, radii (Fig. S1), vertebrae, ilia, sacrum, femora, tibiae, fibula, hand and foot bones. Paleopathological lesions: pseudoarthrosis of left clavicle.

Fig. S1 Radius of individual no 25 used in BMD study

**Individual no. 34 (female, 30-35 years)**


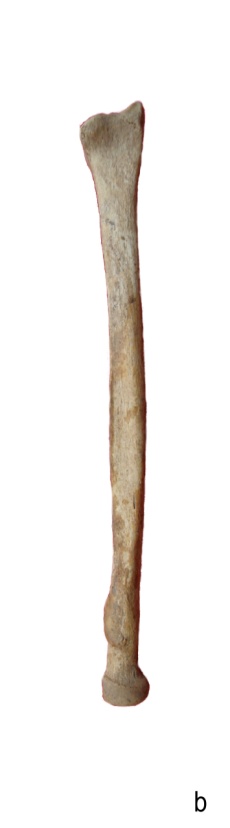
A well-preserved skeleton with a complete skull with slightly damaged maxilla and mandible. The postcranial skeleton was represented by the following bones or their fragments: clavicles, scapula, ribs, humeri, ulnae, radii (Fig. S2), vertebrae, sacrum, pelvis fragments, femora, tibiae, fibulae, patella, hand and foot bones. No paleopathological lesions were observed.

Fig. S2 Radius of individual no 34 used in BMD study

**Individual no. 41 (male, 50-55 years)**


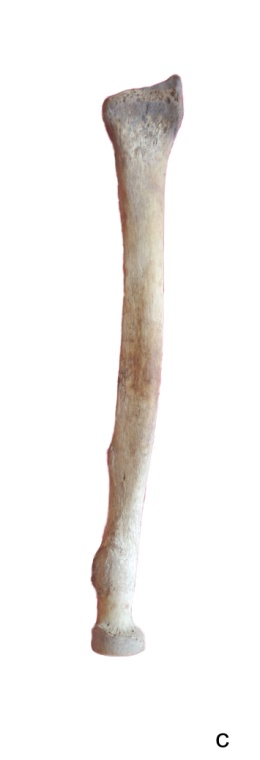
A medium-preserved skeleton with a badly damaged skull. The following skull bones or their fragments were preserved: frontal bone, mandible, few teeth. The postcranial skeleton was represented by the following bones or their fragments: clavicles, scapula, sternum, ribs, vertebrae, sacrum, humeri, ulnae, radii (Fig. S3), pelvis fragments, femora, tibiae, fibula, patellae, hand and foot bones. No paleopathological lesions were observed.

Fig. S3 Radius of individual no 41 used in BMD study

**Individual no. 47 (female, 25-29 years)**

**
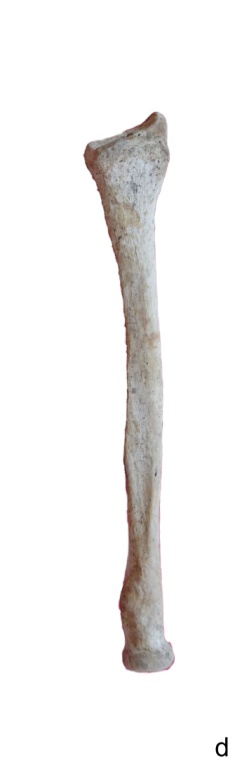
**A poorly-preserved skeleton with a badly damaged skull. The following skull bones or their fragments were preserved: temporal bones, occipital bone, teeth. The postcranial skeleton was represented by the following bones or their fragments: clavicles, scapula, sternum, ribs, vertebrae, sacrum, humeri, ulnae, radii (Fig. S4), ilium, femur, hand and foot bones. No paleopathological lesions were observed.

Fig. S4 Radius of individual no 47 used in BMD study

**Individual no. 63 (male, 25-28 years)**


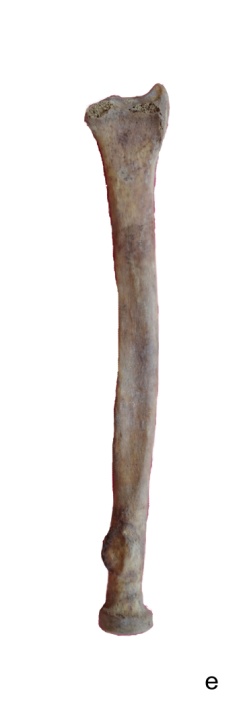
A well-preserved skeleton with completely preserved skull. The postcranial skeleton was represented by the following bones or their fragments: scapulas, sternum, ribs, vertebrae, sacrum, humeri, ulnae, radii (Fig. S5), ilia, femora, tibiae, fibulae, patella, hand and foot bones. No paleopathological lesions were observed.

Fig. S5 Radius of individual no 63 used in BMD study

**Individual no. 64 (male, 43-47 years)**


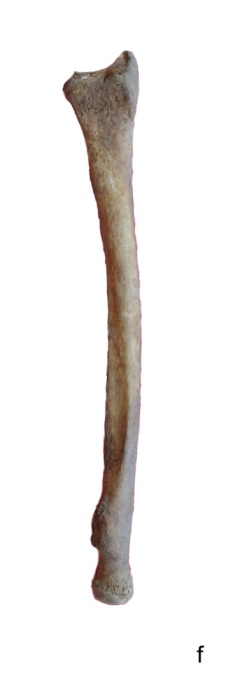
A medium-preserved skeleton with a skull without mandible. The postcranial skeleton was represented by the following bones or their fragments: scapula, ribs, humerus, ulnae, radii (Fig. S6), vertebrae, sacrum, right pelvis bone, femora, tibiae, fibula. Paleopathological lesions: osteophytes, fusion of two lumbar vertebrae, ossified tendons of distal tibia.

Fig. S6 Radius of individual no 64 used in BMD study

**Individual no. 95 (female, 23-26 years)**


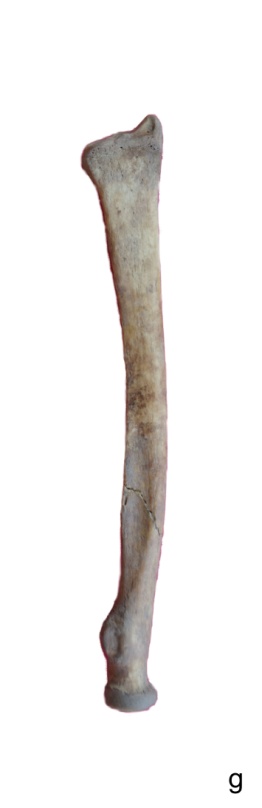
A well-preserved skeleton with a complete skull. The postcranial skeleton was represented by the following bones or their fragments:, scapulas, ribs, humeri, ulnae, radii (Fig. S7), vertebrae, sacrum, tibiae, fibulae, hand and foot bones. Paleopathological lesions: *cribra orbitalia*, tooth hypoplasia, fistula in maxilla.

Fig. S7 Radius of individual no 95 used in BMD study

**Individual no. 96 (male, 40-50 years)**


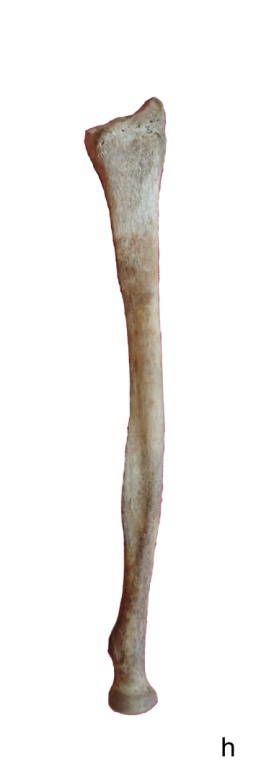
A well-preserved skeleton with a slightly damaged viscerocranium. The postcranial skeleton was represented by the following bones or their fragments: clavicles, scapulas, ribs, humeri, ulnae, radii (Fig. S8), vertebrae, sacrum, pelvis, femora, tibiae, fibulae, patellae, hand and foot bones. No paleopathological lesions were observed.

Fig. S8 Radius of individual no 96 used in BMD study

**Individual no. 99 (male, 40-45 years)**


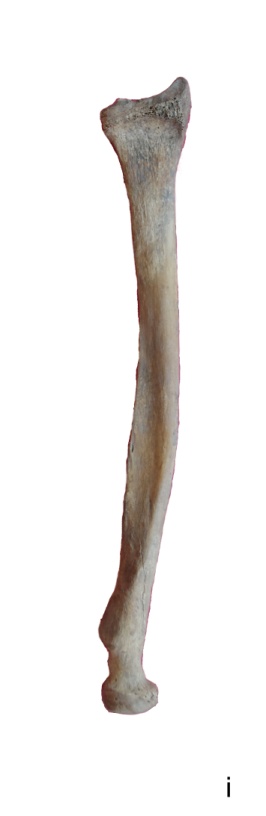
A medium-preserved skeleton with a lack of the neurocranium. From the skull only mandible were preserved. The postcranial skeleton was represented by the following bones or their fragments: clavicle, ribs, ulnae, radii (Fig. S9), vertebrae, sacrum, ilia, femora, hand bones. No paleopathological lesions were observed.

Fig. S9 Radius of individual no 99 used in BMD study

**Individual no. 103 (male, 40-47 years)**

**
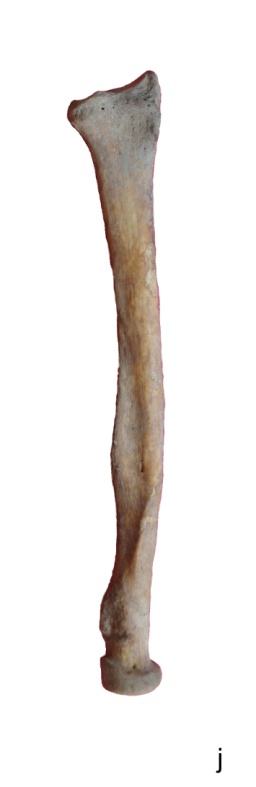
**A medium-preserved skeleton without skull. The postcranial skeleton was represented by the following bones or their fragments: ribs, humeri, ulnae, radii (Fig. S10), vertebrae, sacrum, pelvis bones, femora, tibiae, fibulae, patellae, hand and foot bones. No paleopathological lesions were observed.

Fig. S10 Radius of individual no 103 used in BMD study


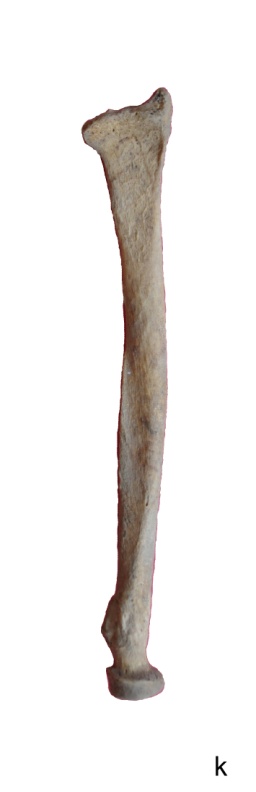
**Individual no. 124 (female, 30-35 years)**

A well-preserved skeleton with a complete skull. The postcranial skeleton was represented by the following bones or their fragments: scapulas, ribs, humeri, ulnae, radii (Fig. S11), vertebrae, sacrum, pelvis bones, tibiae, fibulae, hand and foot bones. No paleopathological lesions were observed.

Fig. S11 Radius of individual no 124 used in BMD study

**Individual no. 125 (female, 24-26 years)**

**
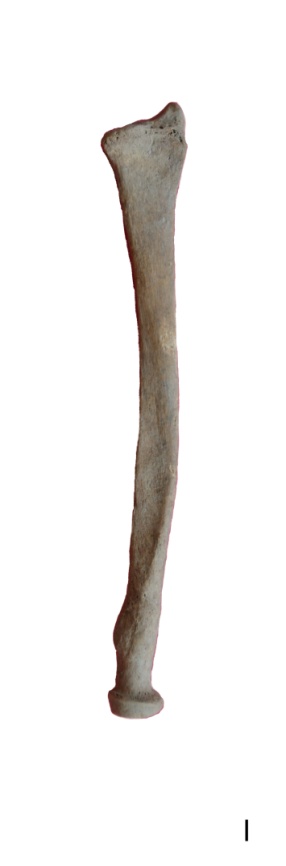
**A well-preserved skeleton with a complete skull. The postcranial skeleton was represented by the following bones or their fragments: clavicles, scapulas, ribs, humeri, ulnae, radii (Fig. S12), vertebrae, sacrum, ilia, femora, tibiae, fibulae, hand and foot bones. Paleopathological lesions: fistula in mandible, tooth hypoplasia.

Fig. S12 Radius of individual no 125 used in BMD study

**Individual no. 130 (female, 28-30 years)**

**
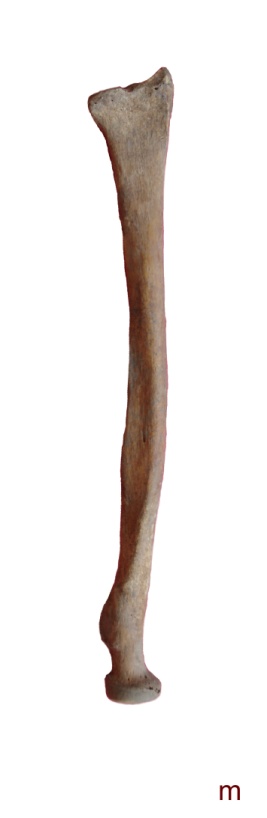
**A medium-preserved skeleton with a complete skull. The postcranial skeleton was represented by the following bones or their fragments: clavicles, scapula, ribs, humerus, ulnae, radii (Fig. S13), vertebrae, sacrum, ilium, hand bones. No paleopathological lesions were observed.

Fig. S13 Radius of individual no 130 used in BMD study

**Individual no. 146 (male, 35-45 years)**

**
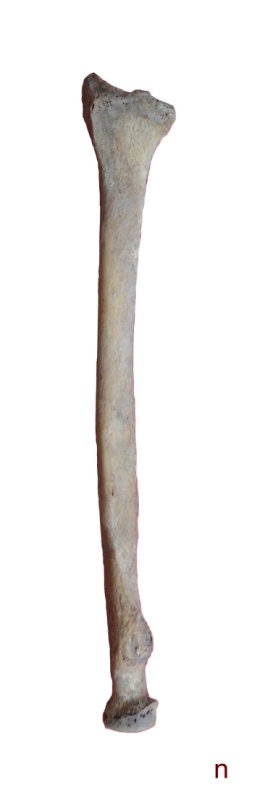
**A medium-preserved skeleton with a complete skull. The postcranial skeleton was represented by the following bones or their fragments: clavicles, scapulas, ribs, humeri, ulnae, radii (Fig. S14), vertebrae, sacrum, ilia, femora, tibiae, fibulae, patellae, hand and foot bones. No paleopathological lesions were observed.

Fig. S14 Radius of individual no 146 used in BMD study

**Individual no. 161 (male, 35-40 years)**


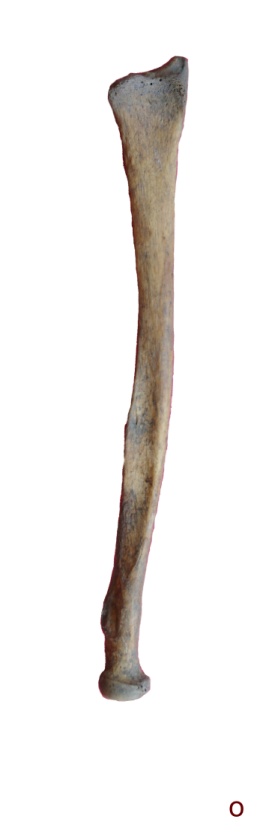
A poorly-preserved skeleton without a neurocranium. One fragment of viscerocranium was present. The postcranial skeleton was represented by the following bones or their fragments: ribs, ulnae, radii (Fig. S15), vertebrae, sacrum, pelvis bones, femora, tibiae, fibulae, patella, hand and foot bones. No paleopathological lesions were observed.

Fig. S15 Radius of individual no 161 used in BMD study

**
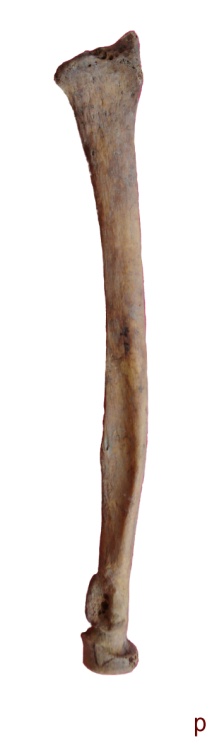
Individual no. 168 (male, 35-40 years)**

A poorly-preserved skeleton without a skull. The postcranial skeleton was represented by the following bones or their fragments: scapulas, sternum, ribs, humeri, radius (Fig. S16), vertebrae, hand and foot bones. Paleopathological lesions: osteophytes on lumbar vertebrae.

Fig. S16 Radius of individual no 168 used in BMD study

**Individual no. 198 (female, 25-30 years)**

**
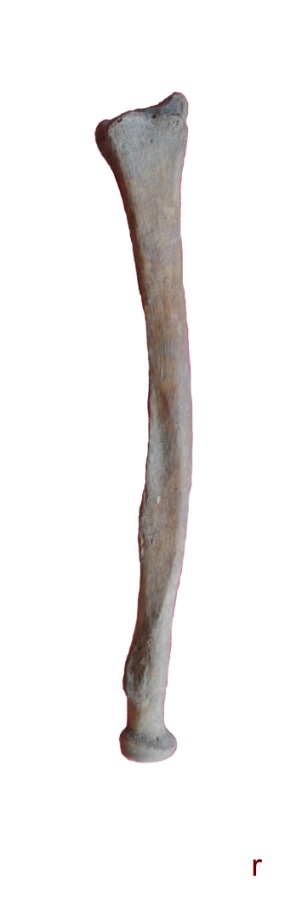
**A well-preserved skeleton with a complete skull. The postcranial skeleton was represented by the following bones or their fragments: clavicles, ribs, humerus, ulnae, radii (Fig. S17), vertebrae, sacrum, pelvis bones, femora, tibiae, fibulae, patella, hand and foot bones. Paleopathological lesions: cribra orbitalia.

Fig. S17 Radius of individual no 198 used in BMD study

**Individual no. 208 (female, 55-60 years)**


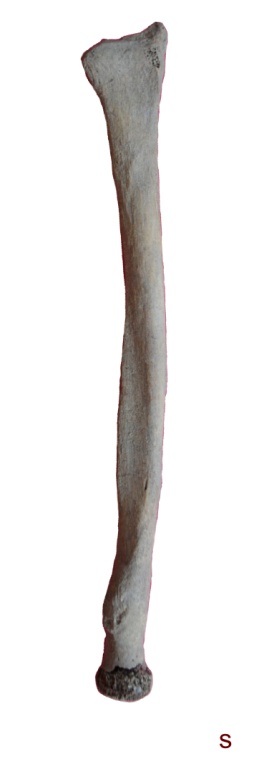
A poorly-preserved skeleton with a badly damaged skull. The postcranial skeleton was represented by the following bones or their fragments: clavicles, scapulas, ribs, humeri, ulnae, radii (Fig. S18), vertebrae, hand and foot bones. Paleopathological lesions: vertebrae shaft compression and osteophytes on the lumbar vertebrae.

Fig. S18 Radius of individual no 208 used in BMD study

**Individual no. 220 (male, 30-35 years)**

**
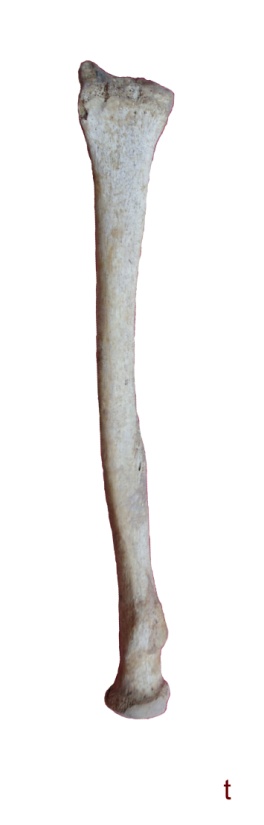
**A poorly-preserved skeleton with a badly damaged skull and broken mandible. The postcranial skeleton was represented by the following bones or their fragments: clavicle, scapula, ribs, humeri, ulna, radii (Fig. S19), vertebrae, pelvis fragments, femora, patellas, hand and foot bones. No paleopathological lesions were observed.

Fig. S19 Radius of individual no 220 used in BMD study

**
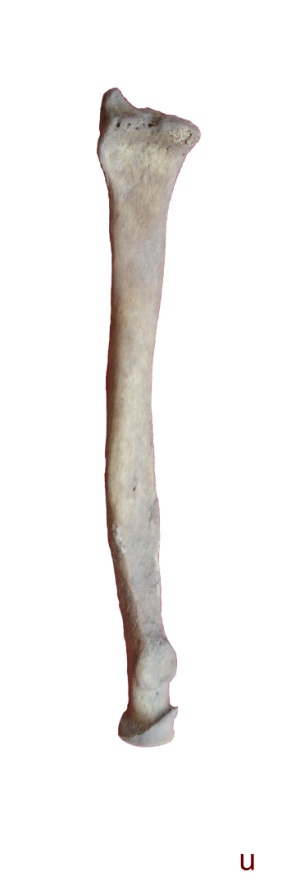
Individual no. 222 (male, 35-40 years)**

A poorly-preserved skeleton without neurocranium but with preserved mandible. The postcranial skeleton was represented by the following bones or their fragments: clavicle, scapulas, ribs, sternum, ulna, radii (Fig. S20), vertebrae, patella, hand bones. No paleopathological lesions were observed.

Fig. S20 Radius of individual no 222 used in BMD study

**Individual no. 243 (female, 30-40 years)**

**
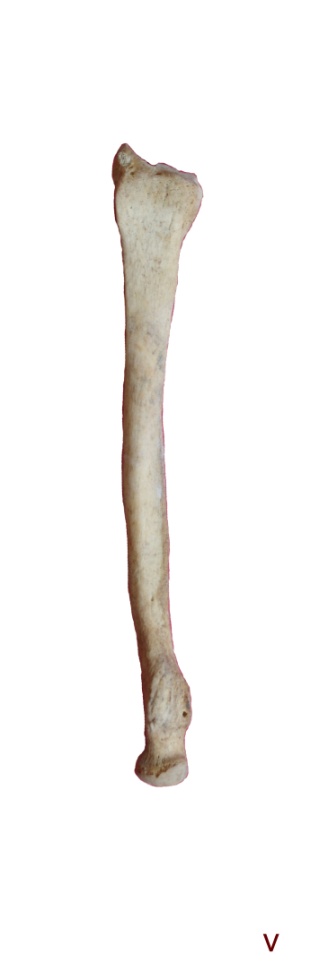
**A well-preserved skeleton with a complete skull. The postcranial skeleton was represented by the following bones or their fragments: clavicles, scapulas, ribs, humeri, ulnae, radii (Fig. S21), vertebrae, sacrum, pelvis bones, femora, tibiae, fibulae, patella, hand and foot bones. Paleopathological lesions: suspicion of osteoporosis.

Fig. S21 Radius of individual no 243 used in BMD study


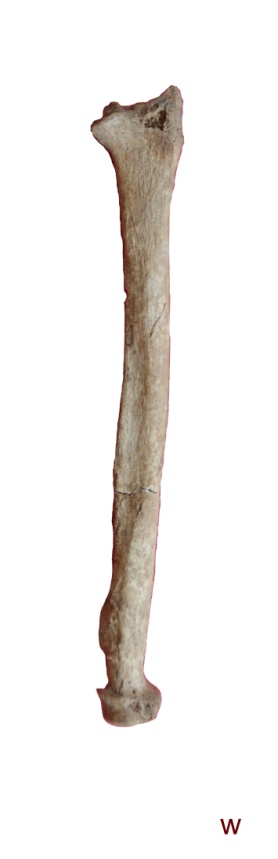
I**ndividual no. 256 (female, 35-44 years)**

A poorly-preserved skeleton without skull. The postcranial skeleton was represented by the following bones or their fragments: clavicles, scapulas, ribs, humeri, ulna, radii (Fig. S22), vertebrae, pelvis fragments. No paleopathological lesions were observed.

Fig. S22 Radius of individual no 256 used in BMD study
